# ENDOCELL-Seud: a Delphi protocol to harmonise methods in endometrial cell culturing

**DOI:** 10.1530/RAF-22-0041

**Published:** 2022-07-28

**Authors:** Andrea Romano, Sun-Wei Guo, Jan Brosens, Asgerally Fazlebas, Caroline E Gargett, Stefan Giselbrecht, Martin Gotte, Linda Griffith, Hugh S Taylor, Robert N Taylor, Hugo Vankelecom, Charles Chapron, Xiao-Hong Chang, Khaleque N Khan, Paola Vigano’

**Affiliations:** 1 Department Obstetrics and Gynecology, GROW – School for Oncology and Reproduction, Maastricht University, Maastricht, The Netherlands; 2 Shanghai Ob/Gyn Hospital, Fudan University, Shanghai, China; 3 Division of Biomedical Sciences, Warwick Medical School University of Warwick, Coventry, UK; 4 Department of Obstetrics, Gynecology & Reproductive Biology, Michigan State University, Grand Rapids, Michigan, USA; 5 The Ritchie Centre, Hudson Institute of Medical Research and Department of Obstetrics and Gynecology, Monash University, Melbourne, Australia; 6 Department of Instructive Biomaterials Engineering, MERLN Institute for Technology-Inspired Regenerative Medicine, Maastricht University, Maastricht, The Netherlands; 7 Department of Gynecology and Obstetrics, Münster University Hospital, Münster, Germany; 8 MIT, Cambridge, Massachusetts, USA; 9 Yale University, New Haven, Connecticut, USA; 10 University at Buffalo, Buffalo, New York, USA; 11 University of Leuven, Leuven, Belgium; 12 Université Paris Cité, Assistance Publique Hôpitaux de Paris, Centre Hospitalier Universitaire Cochin, Department of Gynecology Obstetrics II and Reproductive Medicine, Paris, France; 13 Peking University People’s Hospital, Beijing, China; 14 Department of Obstetrics and Gynecology, The Clinical and Translational Research Center, Graduate School of Medical Science, Kyoto Prefectural University of Medicine, Kyoto, Japan; 15 Fondazione IRCCS Ca’ Granda, Ospedale Maggiore Policlinico, Milano, Italy

**Keywords:** primary endometrial cells, *in vitro* culturing, protocol harmonisation, Delphi method

## Abstract

**In vitro:**

culturing of endometrial cells obtained from the uterine mucosa or ectopic sites is used to study molecular and cellular signalling relevant to physiologic and pathologic reproductive conditions. However, the lack of consensus on standard operating procedures for deriving, characterising and maintaining primary cells in two- or three-dimensional cultures from eutopic or ectopic endometrium may be hindering progress in this area of research. Guidance for unbiased *in vitro* research methodologies in the field of reproductive science remains essential to increase confidence in the reliability of *in vitro* models. We present herein the protocol for a Delphi process to develop a consensus on *in vitro* methodologies using endometrial cells (ENDOCELL-Seud Project). A steering committee composed of leading scientists will select critical methodologies, topics and items that need to be harmonised and that will be included in a survey. An enlarged panel of experts (ENDOCELL-Seud Working Group) will be invited to participate in the survey and provide their ratings to the items to be harmonised. According to Delphi, an iterative investigation method will be adopted. Recommended measures will be finalised by the steering committee. The study received full ethical approval from the Ethical Committee of the Maastricht University (ref. FHML-REC/2021/103). The study findings will be available in both peer-reviewed articles and will also be disseminated to appropriate audiences at relevant conferences.

**Lay summary:**

Patient-derived cells cultured in the lab are simple and cost-effective methods used to study biological and dysfunctional or disease processes. These tools are frequently used in the field of reproductive medicine. However, the lack of clear recommendations and standardised methodology to guide the laboratory work of researchers can produce results that are not always reproducible and sometimes are incorrect. To remedy this situation, we define here a method to ascertain if researchers who routinely culture cells in the lab agree or disagree on the optimal laboratory techniques. This method will be used to make recommendations for future researchers working in the field of reproductive biology to reproducibly culture endometrial cells in the laboratory.

## Introduction

*In vitro* culturing of healthy and diseased cells is widely used to study relevant pathophysiological molecular and cellular events. These methodologies contribute to the understanding of the molecular mechanisms of disease and the identification of therapeutic targets and biomarkers. *In vitro* cultures bear great potential for further development thanks to recent advances in three-dimensional culture systems, co-culturing methods and organ-on-chip technologies (Valdoz *et al.* 2021, Zhou *et al.* 2021, Hammel *et al.* 2022).

Despite the promise of these methodologies, it is widely recognised that studies can be biased when the primary material is not obtained in a standardised manner or rigorously characterised, potentially resulting in reporting of erroneous observations in model systems that do no longer represent the tissue of origin. Along these lines, data from cell culture repositories indicate that a consistent percentage of cultures used for experimental research contain contaminating species or cell types (Romano *et al.* 2020).

A paradigmatic example in the field of reproductive biology is the use of cell cultures established from the endometrium (uterine mucosa) or endometrial implants at ectopic sites, namely endometriosis or adenomyosis (Khan *et al.* 2015, Wang *et al.* 2015, Cook *et al.* 2017, Maybin *et al.* 2017, Lucciola *et al.* 2020, Romano *et al.* 2020, Rawlings *et al.* 2021, Song & Fazleabas 2021, Yu *et al.* 2021). Among other applications, endometrial cell cultures are frequently used to generate *in vitro* models to study the morphological and molecular basis of embryo implantation. Also, primary cells from ectopic implants are commonly employed as preclinical research models of endometriosis or adenomyosis (Zhang *et al.* 2017, Mc Cormack *et al.* 2021).

There is no consensus on standardised operating procedures (SOPs) for obtaining, characterising or culturing primary cells derived from the endometrium or ectopic lesions. There are also no clear quality standards established or recommended in the current literature. Most laboratories use in-house protocols with little or no harmonisation across institutions or laboratories. A recent systematic analysis of the protocols used to isolate primary endometriotic cells showed that most studies do not reference the methods used, while others isolate endometriotic cells using protocols developed for endometrium, which may incompletely recapitulate ectopic lesions (Romano *et al.* 2020). In addition, the cell lineage and the phenotypic characterisations of primary material are frequently inadequate, thus amplifying the risk of contamination by non-endometrial cells or of culturing dedifferentiated endometrial cells that do not retain an endometrial phenotype. In the case of endometriosis, moreover, despite ample evidence suggesting that different disease subtypes have different or even disparate cellular or molecular signatures/functions, most published studies uses a single lesion type that was most conveniently obtained (in most cases, endometrioma) but generalizes the conclusions to all subtypes of endometriosis nonetheless (Romano *et al.* 2020). Consequently, there is poor consistency in the reported findings across studies and a limited confidence in the reliability of *in vitro* models.

To remedy this situation, the ENDOCELL-Seud (indicated as ‘ENDOCELL’ in the rest of the study) project was designed to solicit expert opinions to determine the validity of different methods used to isolate, culture and characterise primary cells from endometrium, endometriosis and adenomyosis and to establish SOPs (Sinha *et al.* 2011). The ENDOCELL project is based on the Delphi method, an iterative investigation method, which, through a series of evaluation stages aims to bring together the most comprehensive opinion, shared in single statements. The tool is commonly used when a consensus on a specific topic needs to be obtained to fill the gap due to the absence of SOPs or guidelines (Coticchio *et al.* 2021, Braungart *et al.* 2022, Hohmann 2022). The ENDOCELL project aims to provide clear guidance to researchers on unbiased *in vitro* methodologies to further the field of reproductive science. The protocol to harness the opinion of experts and to arrive at consensus opinions on the topics debated is described herein.

### Project oversight ENDOCELL Steering Committee

**Figure 1:**
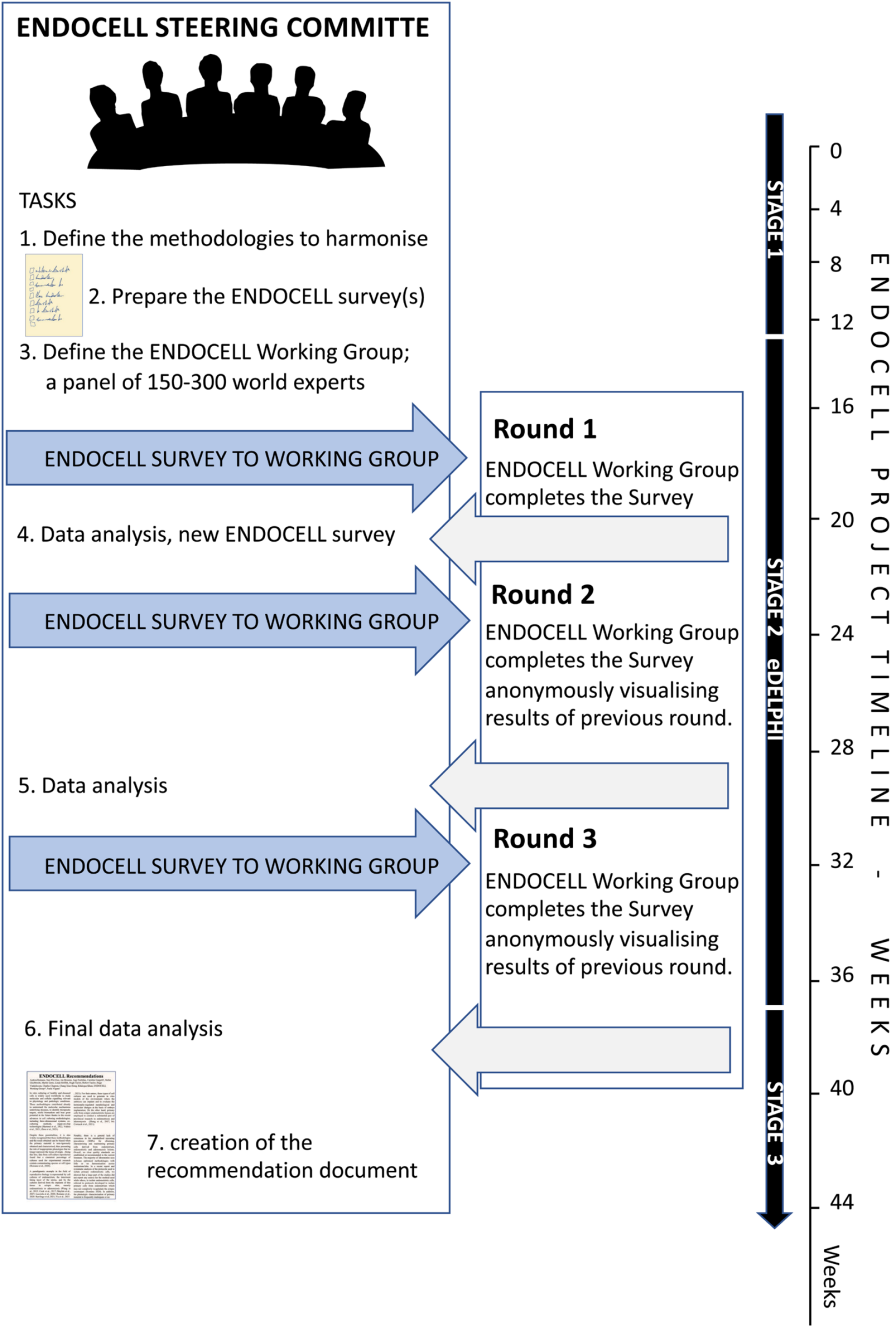
Scheme and timeline of the ENDOCELL project. Workflow of the ENDOCELL project related to an example of 1 of the 11 identified methodologies. Stage 1 will last 12 weeks, during which time the steering committee will define the items to be harmonised related to a specific methodology and will prepare the online ENDOCELL survey. During stage 2, four additional weeks will be necessary to define the ENDOCELL working group specific to the methodology. At week 16, the first eDelphi round will be launched and experts will have 4 weeks to respond (until week 20). Data analyses at the end of each round will take 4 weeks; therefore, the third and last eDelphi round will be completed at week 36 (including data analyses). We expect that within an additional 8 weeks the recommendation documents will be ready. Refer to the main text for additional details.

The project was initiated by the ENDOCELL project coordinator (SWG) and the two facilitators (AR and PV). The project is overseen by the international ENDOCELL steering committee of 12 leading scientists, selected by the coordinator based on publication records and relevant expertise in the field. The ENDOCELL steering committee developed the present protocol, it will follow all phases in the implementation of the protocol and committee members are co-authors of this manuscript. The steering committee works via a series of web-based meetings, which are recorded and can be viewed on demand by committee members. Minutes of the meetings are prepared and circulated. An overview of the ENDOCELL project is outlined in Fig. 1.

### Stage 1. Identification of methodologies and items to harmonise

The first stage in the ENDOCELL project was to define the *in vitro* methodologies in endometrial research needing harmonisation. The ENDOCELL steering committee identified 11 methodologies for which a consensus is lacking, that is, the establishment and maintenance of (1) primary endometrial cells, (2) normal endometrial cell lines, (3) primary endometriotic cells, (4) primary adenomyotic cells, (5) primary myometrial cells, (6) endometriotic cell lines, (7) epithelial organoids, (8) epithelial and stroma organoid (assembloids), (9) spheroids, (10) 3D culture and cell printing, (11) progenitor stem cells, (12) uterine/endometrial-specific endothelial cells and (13) uterine/endometrial-specific immune cells (Table 1). Subsequently, the steering committee will define items in the methodologies that vary across laboratories. The identification and selection of these items will be guided by the expertise of the committee members together with the outcome of a recent systematic review conducted by the ENDOCELL facilitators, which highlighted critical steps in commonly used *in vitro* procedures (Romano *et al.* 2020). In addition, in compliance with similar initiatives in clinical research (Williamson *et al.* 2017) and translational/basic research (Taylor *et al.* 2008, Bustin *et al.* 2009), the following guiding principles will be considered when evaluating items to be included: (i) reporting of the item should facilitate reproducibility of the studies (i.e. readers should be able to replicate the findings based on the information reported such as culture media and supplements), identifying steps in *in vitro* procedures that influence reproducibility; (ii) reporting of the item should facilitate assessment of the quality of the study and risk of bias, allowing avoidance of experimental errors (i.e. temperatures and timing between tissue collection and culture, phenotypic characterisation of cultures, set of required biomarkers and functional features to ensure cell lineage characterisation and if needed subpopulation definition), (iii) a minimum set of items will be determined for reporting in studies employing cell cultures derived from eutopic and ectopic endometrium, ensuring adherence to data governance standards (i.e. requirements of minimal patients’ data).

**Table 1:** Methodologies and items to be harmonised in the ENDOCELL project.

Methodologies	Items	Procedural steps
1. Primary endometrial cells 2. Normal endometrial cell lines 3. Primary endometriosis cells 4. Primary adenomyotic cells 5. Primary myometrial cells 6. Endometriotic cell lines 7. Epithelial organoids 8. Epithelial and stroma organoid (assembloids) 9. Spheroids 10. 3D culture and cell printing 11. Progenitor stem cells 12. Tissue-specific endothelial cells 13. Tissue-specific immune cells	For each methodology, items that varies across labs and introduce risks of bias will be defined. *E.g.: how to dissect primary tissue from patients; what culture media should be used; how to characterise biomarkers, phenotypic and functional features, etc.*	Specific procedural steps related to each item will be defined. *E.g.: Item culture medium: what medium; what supplement; what antibiotics; etc.* This will lead to the development of the ENDOCELL survey

For each item, the steering committee will next identify a series of procedural steps whose definition, reporting and harmonisation are deemed necessary for the guiding principles outlined above to ensure reproducibility, bias assessment and the adoption of minimum standards. For instance, under item ‘culture medium,’ the various kinds of media, supplements, growth factors and antibiotics will be considered (Table 1). Based on the procedural steps identified, the steering committee will create a survey (ENDOCELL survey), consisting of a list of propositions aimed at measuring the consensus for each specific procedural step. For each of the 11 methodologies identified, 1 ENDOCELL survey will be created, with the possibility to combine/merge a number of methodologies into a final single survey.

### Stage 2. Measurement of the consensus across identified methodologies and procedural steps

Consensus will be measured using a three-step Delphi method (Brown *et al.* 2021, Govender 2021), originally designed by RAND corporation in 1950 to create an expert opinion and identify lack of consensus around a specific topic (Claeys *et al.* 2021). The method is based on collecting opinions about specific items from a panel of experts and feeding back anonymously the responses iteratively through several rounds, thus enabling experts to compare the opinions of others together with their own. This method has been extensively used to reach consensus in debated areas of healthcare, ethics, technology and communication. Although variations to the method can be applied, adherence to the Delphi principles is binding: anonymity of responses, iteration and feedback and analysis of the group response. Specifically, for ENDOCELL, we will use an electronic Delphi (eDelphi) method.

### Stage 2a. ENDOCELL surveys

The level of consensus for each procedural step/proposition will be expressed using a 5-point Likert scale (1 = strongly agree, 2 = somewhat agree, 3 = neither agree nor disagree, 4 = somewhat disagree and 5 = strongly disagree), which has already demonstrated consistent outcomes in previous Delphi studies (Akins *et al.* 2005, Vogel *et al.* 2019). A free-text box will be provided for general comments on each item (to justify agreements or suggest wording changes), and a free-text box will be provided at the end of the survey to suggest additional checklist items or provide general comments on the checklist (Colonna *et al.* 2017, Isidori *et al.* 2019). All surveys will be created and administered using Qualtrics (Provo, USA), a user-friendly, feature-rich web-based survey tool that complies to the privacy laws and General Data Protection Regulation.

### Stage 2b. ENDOCELL Working Group (expert panel)

An additional task of the steering committee will be to define the ENDOCELL Working Group, the panel of experts who will be invited to complete the online ENDOCELL survey(s). The ENDOCELL Working Group will be compiled based on a combination of purposive sampling and literature review-based sampling. PubMed will be searched for all papers published in the last 10 years describing the use of cells/*in vitro* systems. Search terms will be (endometriosis OR endometrium) AND (primary cells OR cell line OR organoid OR* in vitro*) NOT (review). This search will be performed prior to launching the ENDOCELL survey specific to each methodology. First and last authors of the papers will be identified as potential members of the ENDOCELL Working Group and duplicate names/authors will be excluded. The generated list of experts will be evaluated by the ENDOCELL steering committee who will prioritise the identified experts according to their suitability in participating to the survey. The steering committee can also add further names. Members of the ENDOCELL Working Group will be recruited from both academic and industrial sectors.

Depending on the methodology/survey, 150–300 ENDOCELL Working Group members will be invited to participate in the survey. Individuals who wish to opt out of the survey will be removed from subsequent invitations. Participants will be blinded to identities of other individuals in the group, and they will not know the specific answers that other individuals provide. The dropout rate based on previous Delphi studies cannot be foreseen in advance given the wide range reported (20–60%) in the literature (Vogel *et al.* 2019, Mendoza *et al.* 2022).

### Stage 2c. Participation to the ENDOCELL survey eDelphi rounds 1, 2 and 3

The ENDOCELL Working Group members will be invited to participate in the survey via a personalised email that explains the objective, the methodology and timelines of this eDelphi protocol. The present protocol (or an extract of it) plus a short video tutorial will also be provided. The ENDOCELL Working Group will be asked to complete three rounds of the eDelphi. They will have a 4-week period to complete each round of the ENDOCELL survey online. In the first eDelphi round, the ENDOCELL Working Group will be also encouraged to add any missing topics/outcomes/questions using the free-text option (Colonna *et al.* 2017, Isidori *et al.* 2019, Vogel *et al.* 2019, Brown *et al.* 2021). The ENDOCELL steering committee will also complete the survey. Each participant in the survey will be assigned a unique identifier to anonymise their responses. Following the first eDelphi round, the ENDOCELL facilitators will analyse the data, identify new items suggested by the ENDOCELL Working Group, and modify the ENDOCELL survey if necessary. The complete ENDOCELL steering committee will be involved in final approval of the new version of ENDOCELL survey that will comprise the identical list of propositions from the previous survey version, plus the newly identified items/procedural steps and corresponding formulated propositions (Fig. 1).

The new ENDOCELL survey will be sent to the ENDOCELL Working Group (eDelphi round 2) together with the results of the first survey round. Results will be visualised in an anonymous manner by each panellist together with their own response from the previous survey round. Participants will be asked to reconsider their responses in light of the anonymised Working group’s responses. Results of the ENDOCELL survey round 2 will be analysed, and a third eDelphi round will be performed (Fig. 1).

### Stage 3. Data analysis and consensus grading

Data will be analysed by the ENDOCELL steering committee at the end of each eDelphi procedure. Results will be reported as median values of the 5-point Likert agreement scale for each procedural step/proposition in the survey. Consensus will be reached for each procedural step/proposition when the sum of choices 1, 2 and 3 (agree) or 4 and 5 (disagree) reaches 70%. No consensus is reached when the sum of the responses for a negative consensus or a positive consensus is <70%.

The strength of agreement will be measured by the mean absolute deviation from the median (MADM), that is, the average distance of the ratings of each participant from the median, and will be ranked as low (MADM > 1.41), moderate (MADM = 1.08–1.41) and high (MADM < 1.08) (Brown *et al.* 2021).

Stability of consensus at the end of the eDelphi procedure will be reached if the between round group responses varied by ≤10%.

### Recommendation document

The ENDOCELL Steering committee will lead the development of an ENDOCELL reporting guidance based on the results of the eDelphi procedure. Two documents will be developed: (1) the statement paper, presenting the agreed recommendations for harmonisation of each item in a specific methodology and (2) an explanation and elaboration paper describing the process and the results. The explanation and elaboration paper will outline the rationale of the reporting items and signalling questions and will provide examples of how to apply the statements. The draft documents will be made available to the members of SEUD (The Society of Endometriosis and Uterine Disorders) and other the scientific organisations like the World Endometriosis Society, European Society of Human Reproduction and Embryology (ESHRE), American Society for Reproductive Medicine (ASRM) and European Endometriosis League (EEL) to both obtain comments and suggestions from the scientific community actively engaged in the field and to obtain endorsement by the respective organisations.

### Timeline

As outlined in Fig. 1, the creation of the recommendation document for each one of the 11 identified methodologies will proceed through 3 defined stages (identification of items to harmonise – stage 1; three-round eDelphi process involving the ENDOCELL working group – stage 2; data analysis and creation of the recommendation document – stage 3) and will take approximately 44 weeks. More than one methodology may be collapsed into a single ENDOCELL survey when items and procedural steps harmonise and show they are analogous. The progress of the complete ENDOCELL project can be seen on the SEUD webpage (to be further defined).

### Dissemination of the ENDOCELL project outcomes

The outcome of the project, the statement and the explanatory papers will be available on the SEUD webpage and will be submitted to peer-reviewed journals for publication. Given that this study is positioned within the ambit of implementation science, the translated study findings will not only be available in peer-reviewed articles but will also be disseminated to appropriate audiences at relevant conferences, like the World Congress of Endometriosis, the annual meetings, workshops or webinars organised by ESHRE, by the ASRM, by the EEL or by SEUD. The Steering committee will (and consensus participants will be encouraged to) publicise the study at key conferences and courses. The knowledge gained from this study will be transferable to other initiatives involving research methodologies by the participants of this particular study. Social media will also be used to disseminate the outcomes. Endorsement of this project by relevant professional societies will be sought, and they may contribute to further disseminating the results of the project.

### Ethical approval

The study received full ethical approval from the Ethical Committee of the Maastricht University (ref. FHML-REC/2021/103). Informed consent will be obtained from all the participants prior to any data collection. There are no anticipated risks to participants who volunteer to participate in the study.

## Declaration of interest

The authors declare that there is no conflict of interest that could be perceived as prejudicing the impartiality of this guideline.

## Funding

This work did not receive any specific grant from any funding agency in the public, commercial or not-for-profit sector.

## Author contribution statement

A R, S W G and P V designed the study. A R sought for ethical approval. A R, S W G and P V supervised the research and writing process. J B, C G, H T, R T, S G, A F, M G, L G, H V, C C, C X-H and K K supervised interpretation and presentation of the study. All authors gave full input to the final draft of the paper.

## References

[bib1] Akins RB Tolson H Cole BR 2005 Stability of response characteristics of a Delphi panel: application of bootstrap data expansion. BMC Medical Research Methodology 5 37. (10.1186/1471-2288-5-37) PMC1318466 16321161

[bib2] Braungart S Williams C Arul SG Bambang K Craigie RJ Cross KM Dick A Hammond P Okoye B Rogers T 2022 Standardizing the surgical management of benign ovarian tumors in children and adolescents: a best practice Delphi consensus statement. Pediatric Blood and Cancer 69 e29589. (10.1002/pbc.29589) 35118808

[bib3] Brown V Moodie M Tran HNQ Sultana M Hunter KE Byrne R Zarnowiecki D Seidler AL Golley R Taylor R 2021 Protocol for the development of core outcome sets for early intervention trials to prevent obesity in children (COS-EPOCH). BMJ Open 11 e048104. (10.1136/bmjopen-2020-048104) PMC8728369 34301658

[bib4] Bustin SA Benes V Garson JA Hellemans J Huggett J Kubista M Mueller R Nolan T Pfaffl MW Shipley GL 2009 The MIQE guidelines: minimum information for publication of quantitative real-time PCR experiments. Clinical Chemistry 55 611–622. (10.1373/clinchem.2008.112797) 19246619

[bib5] Claeys KC Trautner BW Leekha S Coffey KC Crnich CJ Diekema D Fakih MG Goetz MB Gupta K Jones MM 2021 Optimal urine culture diagnostic stewardship practice- results from an expert modified-Delphi procedure. Clinical Infectious Diseases In pressciab987. (10.1093/cid/ciab987) 34849637

[bib6] Colonna P Andreotti F Ageno W Pengo V Marchionni N 2017 Clinical conundrums in antithrombotic therapy management: a Delphi consensus panel. International Journal of Cardiology 249 249–256. (10.1016/j.ijcard.2017.09.159) 28970039

[bib7] Cook CD Hill AS Guo M Stockdale L Papps JP Isaacson KB Lauffenburger DA Griffith LG 2017 Local remodeling of synthetic extracellular matrix microenvironments by co-cultured endometrial epithelial and stromal cells enables long-term dynamic physiological function. Integrative Biology 9 271–289. (10.1039/c6ib00245e) 28317948 PMC5461964

[bib8] Coticchio G Behr B Campbell A Meseguer M Morbeck DE Pisaturo V Plancha CE Sakkas D Xu Y D’Hooghe T 2021 Fertility technologies and how to optimize laboratory performance to support the shortening of time to birth of a healthy singleton: a Delphi consensus. Journal of Assisted Reproduction and Genetics 38 1021–1043. (10.1007/s10815-021-02077-5) 33599923 PMC8190195

[bib9] Govender P 2021 Identifying and bridging the knowledge-to-practice gaps in rehabilitation professionals working with at-risk infants in the public health sector of South Africa: a multimethod study protocol. BMJ Open 11 e039242. (10.1136/bmjopen-2020-039242) PMC8137249 34006535

[bib10] Hammel JH Zatorski JM Cook SR Pompano RR Munson JM 2022 Engineering in vitro immune-competent tissue models for testing and evaluation of therapeutics. Advanced Drug Delivery Reviews 182 114111. (10.1016/j.addr.2022.114111) PMC8908413 35031388

[bib11] Hohmann E 2022 Editorial commentary: wider acceptance of medical expert consensus research requires strict adherence to Delphi panel methodology. Arthroscopy 38 250–252. (10.1016/j.arthro.2021.08.014) 35123707

[bib12] Isidori AM Giammusso B Corona G Verze P 2019 Diagnostic and therapeutic workup of erectile dysfunction: results from a Delphi consensus of andrology experts. Sexual Medicine 7 292–302. (10.1016/j.esxm.2019.04.001) 31196744 PMC6728771

[bib13] Khan KN Kitajima M Hiraki K Fujishita A Nakashima M Masuzaki H 2015 Decreased expression of human heat shock protein 70 in the endometria and pathological lesions of women with adenomyosis and uterine myoma after GnRH agonist therapy. European Journal of Obstetrics, Gynecology, and Reproductive Biology 187 6–13. (10.1016/j.ejogrb.2015.01.012) 25697974

[bib14] Lucciola R Vrljicak P Gurung S Filby C Darzi S Muter J Ott S Brosens JJ Gargett CE 2020 Impact of sustained transforming growth factor-beta receptor inhibition on chromatin accessibility and gene expression in cultured human endometrial MSC. Frontiers in Cell and Developmental Biology 8 567610. (10.3389/fcell.2020.567610) PMC7490520 32984350

[bib15] Maybin JA Thiruchelvam U Madhra M Saunders PTK Critchley HOD 2017 Steroids regulate CXCL4 in the human endometrium during menstruation to enable efficient endometrial repair. Journal of Clinical Endocrinology and Metabolism 102 1851–1860. (10.1210/jc.2016-3604) 28323919 PMC5470763

[bib16] Mc Cormack B Maenhoudt N Fincke V Stejskalova A Greve B Kiesel L Meresman GF Vankelecom H Götte M Barañao RI 2021 The ellagic acid metabolites urolithin A and B differentially affect growth, adhesion, motility, and invasion of endometriotic cells in vitro. Human Reproduction 36 1501–1519. (10.1093/humrep/deab053) 33748857

[bib17] Mendoza HF Hobson S Windrim RC Kingdom J Rojas-Gualdron D 2022 Identification of Eessential steps in outlet forceps-assisted vaginal delivery: a Delphi study. Journal of Obstetrics and Gynaecology Canada 44 675–682. (10.1016/j.jogc.2022.01.008) 35074484

[bib18] Rawlings TM Makwana K Taylor DM Mole MA Fishwick KJ Tryfonos M Odendaal J Hawkes A Zernicka-Goetz M Hartshorne GM 2021 Modelling the impact of decidual senescence on embryo implantation in human endometrial assembloids. eLife 10 e69603. (10.7554/eLife.69603) PMC8523170 34487490

[bib19] Romano A Xanthoulea S Giacomini E Delvoux B Alleva E Vigano P 2020 Endometriotic cell culture contamination and authenticity: a source of bias in in vitro research? Human Reproduction 35 364–376. (10.1093/humrep/dez266) 32106286 PMC7048714

[bib20] Sinha IP Smyth RL Williamson PR 2011 Using the Delphi technique to determine which outcomes to measure in clinical trials: recommendations for the future based on a systematic review of existing studies. PLoS Medicine 8 e1000393. (10.1371/journal.pmed.1000393) PMC3026691 21283604

[bib21] Song Y Fazleabas AT 2021 Endometrial organoids: a rising star for research on endometrial development and associated diseases. Reproductive Sciences 28 1626–1636. (10.1007/s43032-021-00471-z) 33533008

[bib22] Taylor CF Field D Sansone SA Aerts J Apweiler R Ashburner M Ball CA Binz PA Bogue M Booth T 2008 Promoting coherent minimum reporting guidelines for biological and biomedical investigations: the MIBBI project. Nature Biotechnology 26 889–896. (10.1038/nbt.1411) PMC2771753 18688244

[bib23] Valdoz JC Johnson BC Jacobs DJ Franks NA Dodson EL Sanders C Cribbs CG Van Ry PM 2021 The ECM: to scaffold, or not to scaffold, that is the question. International Journal of Molecular Sciences 22 12690. (10.3390/ijms222312690) PMC8657545 34884495

[bib24] Vogel C Zwolinsky S Griffiths C Hobbs M Henderson E Wilkins E 2019 A Delphi study to build consensus on the definition and use of big data in obesity research. International Journal of Obesity 43 2573–2586. (10.1038/s41366-018-0313-9) 30655580 PMC6892733

[bib25] Wang X Mamillapalli R Mutlu L Du H Taylor HS 2015 Chemoattraction of bone marrow-derived stem cells towards human endometrial stromal cells is mediated by estradiol regulated CXCL12 and CXCR4 expression. Stem Cell Research 15 14–22. (10.1016/j.scr.2015.04.004) 25957946 PMC5001152

[bib26] Williamson PR Altman DG Bagley H Barnes KL Blazeby JM Brookes ST Clarke M Gargon E Gorst S Harman N 2017 The COMET Handbook: version 1.0. Trials 18 280. (10.1186/s13063-017-1978-4) PMC5499094 28681707

[bib27] Yu J Berga SL Meng Q Xia M Kohout TA Van Duin M Taylor RN 2021 Cabergoline stimulates human endometrial stromal cell decidualization and reverses effects of interleukin-1beta in vitro. Journal of Clinical Endocrinology and Metabolism 106 3591–3604. (10.1210/clinem/dgab511) 34260712 PMC8864758

[bib28] Zhang Q Dong P Liu X Sakuragi N Guo SW 2017 Enhancer of Zeste homolog 2 (EZH2) induces epithelial-mesenchymal transition in endometriosis. Scientific Reports 7 6804. (10.1038/s41598-017-06920-7) PMC5533797 28754964

[bib29] Zhou Z Zhu J Jiang M Sang L Hao K He H 2021 The combination of cell cultured technology and in silico model to inform the drug development. Pharmaceutics 13. (10.3390/pharmaceutics13050704) PMC8151315 34065907

